# Perceptions and experiences of childhood vaccination communication strategies among caregivers and health workers in Nigeria: A qualitative study

**DOI:** 10.1371/journal.pone.0186733

**Published:** 2017-11-08

**Authors:** Afiong Oku, Angela Oyo-Ita, Claire Glenton, Atle Fretheim, Heather Ames, Artur Muloliwa, Jessica Kaufman, Sophie Hill, Julie Cliff, Yuri Cartier, Eme Owoaje, Xavier Bosch-Capblanch, Gabriel Rada, Simon Lewin

**Affiliations:** 1 Community Medicine Department, University of Calabar, Calabar Municipality, Cross River State, Nigeria; 2 Norwegian Institute of Public Health, Oslo, Norway Boks, St Olavs plass, Oslo, Norway; 3 Institute of Health and Society, University of Oslo, Blindern, Oslo, Norway; 4 Direccão Provincial de Saude de Nampula, Departamento de Saude, Nampula-Mozambique; 5 Centre for Health Communication and Participation School of Psychology and Public Health La Trobe University Victoria, Australia; 6 Faculdade de Medicina, Universidade Eduardo Mondlane, Maputo, Mozambique; 7 International Union for Health Promotion and Education, Saint-Maurice, France; 8 Community Medicine Department, University of Ibadan Oyo state, Nigeria; 9 Swiss Tropical and Public Health Institute, Basel, Switzerland; 10 University of Basel, Basel, Switzerland; 11 Evidence-based Healthcare Programme, Pontificia Universidad Catolica de Chile, Santiago, Chile; 12 Health Systems Research Unit,South African Medical Research Council, Cape Town, South Africa; University of Washington, UNITED STATES

## Abstract

**Background:**

Effective vaccination communication with parents is critical in efforts to overcome barriers to childhood vaccination, tackle vaccine hesitancy and improve vaccination coverage. Health workers should be able to provide information to parents and other caregivers and support them in reaching decisions about vaccinating their children. Limited information exists regarding the perceptions of caregivers and health workers on the vaccination communication strategies employed in Nigeria. This study, which forms part of the ‘Communicate to vaccinate’ (COMMVAC) project, aims to explore the perceptions and experiences of caregivers and health workers in Nigeria on vaccination communication strategies implemented in their settings.

**Methodology:**

We conducted the study in two States: Bauchi in Northern Nigeria and Cross River in the south. We carried out observations (n = 40), in-depth interviews (n = 14) and focus group discussions (FGDs) (n = 12) amongst 14 purposively selected health workers, two community leaders and 84 caregivers in the two states. We transcribed data verbatim and analysed the data using a framework analysis approach.

**Results:**

Caregivers were informed about vaccination activities through three main sources: health facilities (during health education sessions conducted at antenatal or immunization clinics); media outlets; and announcements (in churches/mosques, communities and markets). Caregivers reported that the information received was very useful. Their preferred sources of information included phone text messages, town announcers, media and church/mosque announcements. Some caregivers perceived the clinic environment, long waiting times and health worker attitudes as barriers to receiving vaccination information.When delivering communication interventions, health workers described issues tied to poor communication skills; poor motivation; and attitudes of community members, including vaccine resistance.

**Conclusion:**

Communication about vaccination involves more than the message but is also influenced by the environment and the attitudes of the deliverer and receiver. It is pertinent for health policy makers and programme managers to understand these factors so as to effectively implement communication approaches.

## Introduction

Immunization remains one of the most successful and cost-effective public health interventions for disease prevention[1].It has been estimated that the Expanded Programme on Immunization (EPI) in low and middle-income countries (LMICs) has prevented more than two million child deaths since its initiation in 1974 [2].Although, Africa has made some progress in immunization services, large numbers of children remain unvaccinated and under-vaccinated. Only a quarter of eligible children in Nigeria receive all recommended vaccinations[3].This is well below the 90% level of coverage recommended by the World Health Organization (WHO) for the sustained control of vaccine-preventable diseases (VPDs)[4].

Some of the reasons for Nigeria’s consistently low immunization coverage rates include mothers’ poor knowledge of immunization, leading to low confidence and lack of trust[5];concerns about immunization safety; long distances to and long waiting times at health facilities; and poor attitudes and skills of health workers[6–9]. Some of these problems are linked to gaps in communicating vaccination information. Several studies suggest that parents’ good understanding of vaccine-preventable diseases, how vaccination works and the vaccination schedule contribute to children being vaccinated[6, 10, 11]. Although evidence of the effectiveness of communication interventions in improving vaccination uptake is limited and mixed[12, 13], effective communication with parents is likely to be a key factor in improving vaccination outcomes [14, 15].

Health workers have a central role in maintaining public trust in vaccination programmes. Effective interaction between parents and health workers could address the concerns of vaccine-supportive parents and motivate a hesitant parent towards vaccine acceptance[16–18]. Conversely, poor communication can contribute to rejection of vaccinations or dissatisfaction with care [19, 20]. Furthermore, poor health worker communication and engagement with community leaders and groups have been linked to poor understanding of routine immunization schedules and the specific diseases they target[21].

This study forms part of the ‘Communicate to vaccinate 2’(COMMVAC 2) project, an international project exploring how to integrate evidence-based communication strategies that are adapted for local conditions into vaccination programmes in selected LMICs. Previous papers have looked at the range of communication interventions being used[22, 23]and health systems level factors affecting vaccination communication[24]. In this paper, we focus on health workers’ and caregivers’ perceptions of communication strategies in Nigeria.

### Study objectives

1. To explore perceptions and experiences of caregivers and health workers in Nigeria towards childhood vaccination communication
2. To explore caregivers’ and health workers’ views regarding preferred channels or modes of information on childhood vaccination

## Materials and methods

### Study area

Our study setting was Nigeria, the most populous country in Africa, with an estimated population of over 160 million people. Nigeria currently has 36 states and the Federal Capital Territory, Abuja. These States are further divided into local government areas, and each local government area is divided into wards. The Nigeria people are multi-ethnic, multicultural and multi-religious.

The National Primary Health Care Development Agency is responsible for the control of VPDs in Nigeria through vaccine provision and immunization guidelines. At the national level, responsibility for the development of communication interventions for vaccination programmes is given to the National Social Mobilization Working Group, while State and Local Social Mobilization Committees are responsible for coordinating and implementing communication interventions. The national immunization schedule recommends that all routine childhood vaccinations be completed by nine months of age. Apart from the routine immunization schedule, several rounds of supplementary/mass campaigns are also held across the country every year.

### Study sites

We carried out the study in rural and urban settings in two States: Cross River in southern Nigeria and Bauchi in northern Nigeria. We selected these two States based on variations in vaccination coverage rates, with rates being lower in Bauchi than in Cross River (DPT3 coverage rates of 12.5% and 76.1% respectively)[3]; and variations in terms of vaccine hesitancy, with vaccine refusal rates being much higher in Bauchi, linked to religious and cultural beliefs[25].Furthermore, at the time of the study, Bauchi was one of the12 polio-prevalent States of Northern Nigeria and had been the focus of global and national efforts to eliminate polio compared to Cross River which has received less attention. We selected Cross River State to provide an example of a good performer in terms of vaccination coverage, with a coverage rate of 52.5% (16). In addition, Cross River State has remained polio-free for the last decade. Lastly, the two states also differ in terms of religion, with the people of Bauchi being predominantly Muslim while those in Cross River are predominantly Christian.

### Study population

We employed purposive sampling to recruit participants for the study. With the help of facility health workers, we invited caregivers (here defined as both parents and other caregivers of children) who brought children to facilities for vaccination. In the urban sites in both States, we selected caregivers who could communicate in English or Pidgin English. For the rural sites, we selected caregivers who could communicate in the local languages of the community.

Caregivers were between 18–55 years old. In the rural areas in both states, the caregivers were mainly petty traders, farmers or housewives with at least primary education. In the urban sites of both states, most caregivers had at least secondary education and were mainly civil servants, teachers, students, traders and housewives.

For health workers, we purposively selected those who had received training in vaccination delivery; who had been involved in vaccination activities for at least six months; and who played a major role in communicating information regarding vaccination to caregivers. The health workers were mainly nurses, community health officers and community health extension workers.

Finally, with the help of the health workers, we recruited one traditional leader and one religious leader who were actively involved in the delivery of vaccination services in Bauchi State. We included traditional and religious leaders in Bauchi state only because these leaders are actively involved in a range of vaccination related activities. In contrast, traditional and religious leaders in Cross River state were only engaged during campaigns and therefore had a much more limited role.

We received consent from all respondents before we conducted the interviews.

### Data collection methods

#### Focus group discussions with caregivers

The FGDs with caregivers included semi-structured questions that covered:

- Their sources of information regarding childhood vaccination
- The type of information they received
- Their perceptions of these communication strategies
- Their views of the best possible ways in which vaccination information should be delivered

Each focus group was made up of six to eight persons, a moderator, a note taker and an observer. We conducted the FGDs in primary health care centres on routine immunization clinic days in order to capture caregivers’ experiences of communication interventions provided during campaigns and routine services in the community and in facilities. Participants were seated around a round table to ensure proper eye contact and attention. The moderator, who was the principal investigator, facilitated the discussion and ensured equal participation. We obtained permission from participants to record the discussion. In Cross River, we conducted the FGDs mainly in pidgin English and Efik. In Bauchi, we conducted the FGDs in Hausa. We engaged an interpreter to assist with communication. Each discussion lasted about 30–45 minutes. The number of focus group discussions held was guided by practical constraints such as the amount of time available for data collection. We conducted interviews to cover urban and rural respondents and the number of group discussions was determined by the number of service users present in the health facility.We provided refreshments for participants at the end of each discussion.

#### In-depth interviews with health workers

We invited the health workers for an interview at a time and date convenient for them.We aimed to interview two health workers per facility, the head of the facility and a health worker actively involved in providing vaccination services. However, in certain rural facilities only one health worker was seen manning all the activities in the clinic. We carried out the individual interviews in English, in the health facility, using an interview guide (SF2). Each interview lasted between 30–45 minutes and were audio-recorded after permission was obtained from the respondents.

#### Interviews with traditional and religious leaders

We conducted interviews with one religious and one traditional leader in Bauchi State who were actively involved in the vaccination programme.

#### Observations of communication activities

We carried out observations of routine immunization activities in six health facilities in rural and urban settings in both states over a period of four weeks. This was done after obtaining permission from the health worker in charge of each facility. We recruited three research assistants who could speak Efik and Hausa fluently and conducted a two-day training workshop for them. During the first week of observations, we had daily meetings with the research assistants to ensure that they were collecting accurate information. The research assistants conducted structured observations of one-to-one communication between health workers and caregivers during routine vaccination encounters; and of group health education sessions conducted on vaccination days.

We observed the following during our visits:

- The content of the communication interventions
- The frequency with which it was delivered and the format(s) used
- Who delivered the intervention
- Whom the communication intervention targeted
- Whether the communication intervention included information on other childhood interventions (e.g. nutrition breast feeding, diarrhoea management, etc.)
- The duration of each session

During the polio campaigns, held in both states, we also accompanied health workers during house visits in communities and observed how they communicated with the caregivers.

#### Data management and analysis

We audio-recorded and transcribed verbatim all interviews and FGDs. Data that we collected in Hausa and Efik were translated into English. Two researchers (AO and GB) carried out data analysis using a framework analysis approach. It involved four steps: familiarization, indexing/coding, charting, and mapping/interpretation[26].First, we familiarized ourselves with the data collected in the interviews and the FGDs by listening repeatedly to the audio recordings and studying the transcripts. This helped us gain an overview of the body of material gathered and become aware of key ideas as well as recurrent themes. Next, we indexed and coded the data. To enhance coding validity, the principal investigator (AO) and a sociologist(GB)coded each transcript independently and later merged the codes to create a single coding book. When coding the data, we identified a number of themes, which we then organized under different sub-categories and categories in a chart which we subsequently populated with our textual data. Lastly, in the mapping and interpretation stage, we analysed the key characteristics as laid out in the charts, looked at patterns across and within the data and made preliminary explanations of associations with the data. We then used relevant data extracts to demonstrate key findings.

For the observations, we examined the observation notes and tried to identify patterns in behaviour and possible factors that could impede or enhance vaccination communication at the facility level. We then tried to link the findings from the observations to the findings from the interviews and FGDs, which helped to confirm or refute these findings.

#### Ethical approval

We sought and obtained ethical approval from the Cross River State Health Research and Ethics Committee and Bauchi State Health Research and Ethics Committee. The Regional Committee for Medical Research Ethics in Norway assessed the project as not requiring ethical approval under the Norwegian Act on Medical and Health Research.

## Results

We conducted the study between April and July 2014. We held 12 focus group discussions (FGDs), four in rural and eight in urban sites. These discussions included 84 caregivers (78 mothers, four grandmothers and two aunts). We also carried out 14 interviews with vaccinators (six rural and eight urban) and two interviews with one traditional and one religious leader in Bauchi State. Finally, we carried out 40 observations of communication activities for vaccination in selected urban (24) and rural (16) sites in both states (Table 1).

**Table 1:** Rural-urban distribution of focus group discussions, interviews and observation of respondents.

STATE	RESPONDENTS	RURAL	URBAN	TOTAL
Cross River	Focus group discussions with caregivers	2	6	8
	Interviews with vaccinators	4	4	8
	Observations	8	16	24
Bauchi	Focus group discussions with caregivers	2	2	4
	Interviews with vaccinators	3	3	6
	Traditional leader	1	-	1
	Religious leader	1	-	1
	Observations	8	8	16

### Caregivers’ current sources of information regarding vaccination

In both Bauchi and Cross River States, caregivers mentioned that the health workers at the health facilities were their main source of information regarding childhood vaccination during antenatal clinics. Caregivers also reported that they received vaccination information from the media (radio and television jingles, announcements).Radio was more commonly mentioned by rural caregivers and television by their urban counterparts. Caregivers in rural settings also commonly referred to town announcers, church announcements, sensitization in markets and home visits by health workers. A few caregivers mentioned that they heard about vaccination from older women in the community or from their friends and neighbours.

### Content of information in health education sessions received by/delivered to caregivers in vaccination clinics

All caregivers expressed that the messages received in the clinic were useful. All caregivers reported that they received information about managing vaccination side effects in both states. However, most caregivers in Cross River State reported that most of the information they received from health workers in clinics focused on other child health strategies (nutrition, childcare, personal hygiene) and reported a paucity of information on vaccination. We observed that in most urban facilities in Cross River State, vaccination-related messages varied in content, depending on the deliverer of the session. In addition, the content was more in-depth when mothers of newborns visited the facility or just before a campaign. Based on our observations, the health workers in the rural sites provided basic information on vaccination in the local language and this information was usually reinforced just before campaigns with an emphasis on the need for parents to present their children for vaccination. Health talks in rural facilities were not as detailed as those given in the urban health facilities.

In contrast, in Bauchi State, vaccination messages dominated the health education sessions, with an emphasis on acceptance of polio vaccines. Caregivers were educated on the need to have children fully vaccinated, the benefits, side effects and the immunization schedule. Based on our observations, the urban facilities provided more detailed information compared to the rural sites, where facilities were often short-staffed.

### Caregivers’ perceptions of communication in vaccination clinics

#### a) Clinic environment

Most caregivers agreed that the clinic environment where vaccination messages were received played an important role in the delivery of vaccination information. More caregivers in both states reported that the environment was not conducive. Dissatisfied caregivers complained that the number of seats was not adequate and that they sometimes had to stand throughout the health talks or remain outside as the clinic could not accommodate them. This led to caregivers either missing the health talks or not paying attention to, or participating fully, during the talks.

*“Because the ventilation in the health centre is poor*, *it gets very stuffy when there is a crowd*, *especially when first time mothers visit*. *Most babies become uncomfortable and start crying*. *Then their mothers have to carry them outside for fresh air and in the process may miss vital information about vaccination and child care*.*”*(Caregiver, Cross River, urban site.)

#### b) Long waiting times

Most caregivers in both states agreed that they had to spend a considerable amount of time in the health centre when bringing their children for vaccination. Caregivers described this as having both positive and negative effects. Some respondents, especially in Bauchi, were particularly happy about spending time in the clinic because it afforded them the opportunity to ask questions or share experiences, not just about vaccination but about childcare in general.

*“The time I spend in the health centre is valuable to me so I don’t mind staying for a long time there*. *Apart from learning from the health talks given by the health professionals*, *I also use that time to learn from other mothers’ experiences and they also give me ideas on how to deal with some health issues*. *So*, *I will not say it is time wasted*. *For example*, *the health workers advise us to give paracetamol after the baby is given the penta vaccine but I realize my child still has high fever and is restless the whole night*. *But one mother advised that I should administer paracetamol before coming to the clinic and suggested a particular brand which I bought*, *did as she advised and the fever vanished*.*”*(Caregiver, Bauchi, urban site.)

This was confirmed during the observations where caregivers met with other women or health workers and interacted, exchanged ideas, asked questions and learnt from one another while waiting for the clinic to commence. However, urban caregivers in both states complained that clinics commenced very late (10 AM)and went on for too long, usually closing at 2–3 PM.

Caregivers mentioned that they received talks from different people on various topics of interest, including the representative of a baby diaper company. Some caregivers believed that if the clinic started early and the information given was concise it would be more useful than starting late. They mentioned that most babies become irritable later in the day, leading mothers to pay less attention during the health talk. Another reason for long waits in rural sites was the delay in arrival of other mothers at the health centre. They explained that most caregivers usually came late to the clinic and the health workers would usually not commence until a sufficient number of mothers had arrived.

*“I usually prefer coming to the clinic from 10 AM because the health workers usually like wasting time*. *So*, *I prefer going to my shop first and sorting things out before coming to the health centre*. *Because of this*, *I usually miss the health talks where I learn how to take good care of my baby and receive information on the vaccinations given to him*. *I wish they could start on time so that we can leave early and get back to our businesses*.*”*(Caregiver, Cross River, urban site.)

We also observed that long waiting periods in the rural sites was a result of only one health worker being in charge of all clinic activities. One healthworker also gave reasons for the long waiting times experienced by caregivers:

*“This is because we have to go to the cold chain store to collect the vaccine every day because our solar system [power supply] has a problem*. *Before now*, *we would collect it a day before and store it in our clinic*. *But because our solar system is bad we collect the vaccines on the day we want to use it and this causes a delay in the early commencement of the clinic*. *Also when we are expecting visitors (representatives from WHO*, *NGOs) to come we have to delay the clinic from starting until the visitors come*.*”*(Health worker, Bauchi, urban site.)

#### c) Heath worker attitudes towards caregivers

In both states, caregivers had varying opinions regarding the way in which health workers communicated with them. Some described health workers as being warm and friendly and treating them with respect. However, a few caregivers described the impolite behaviour of health workers towards women with low levels of education, teenage mothers and mothers who arrived late or forgot their vaccination cards. They explained that this behaviour could undermine trust in the health workers and could also discourage caregivers from listening to health education messages. One caregiver explained that once a mother is treated inappropriately, she may become resistant to any information delivered by the health worker and may resolve not to return to the health facility to continue with her child’s vaccination:

*“If you don’t dress well and are not so educated some of the nurses treat you badly compared to others*. *This attitude usually leaves a lasting impression on the minds of the mother and she may miss vital information on vaccination because she refuses to listen to anything the health workers say*. *I almost fought with one of them [nurses] the last time I visited because of this behaviour*.*”* (Caregiver, Bauchi, rural site.)

Some caregivers also reported that health workers gave preference to some women over others:

*“I have noticed that the health workers don’t operate a first-come first-served basis and are usually partial to some mothers*. *You can get to the clinic very early but you may be the last to leave because if the health worker’s friend arrives*, *she will be given preferential treatment while others were abandoned*. *This usually makes some mothers stay at home or come late and miss important information delivered during the teaching session and some may never complete their wards vaccination*.*”*(Caregiver, Cross River, urban site.)

This was confirmed during our observation visits during which we noted that caregivers who dropped off their vaccination cards and were registered early were not usually the first to receive vaccinations.

We observed that health workers were particularly warm and receptive on days when fewer caregivers visited. Health workers occasionally shouted at mothers who came late, could not answer questions asked, missed their clinic appointments or forgot their vaccination cards at home. In the rural sites, our observations also suggested that these health workers were usually not as friendly as urban health workers. This may have been tied to the fact that they were understaffed, with sometimes just one health worker in a facility performing tasks including the collection of vaccines from the central store, registration, vaccine administration, health education and cleaning. We rarely observed one-to-one communication in these rural settings. Only a few caregivers asked questions following a health education session while others were too timid. We observed that caregivers, especially latecomers, did not to go to the health workers to ask questions or to seek clarifications on their children’s health needs because of fear of being scolded. Rather we observed that they usually sought information from other caregivers at the clinic.

### Caregivers’ preferences regarding information on childhood vaccination

#### Caregivers’ preferred channels of childhood vaccination information

In both states, a vast majority of urban caregivers reported that they preferred to receive vaccination information via text messages, including reminders of vaccination clinic appointments or upcoming campaigns. Most rural caregivers, on the other hand, wanted childhood vaccination information delivered to them through the town announcers. They suggested that town announcers should be engaged continuously to disseminate information, rather than during campaigns only. The media was a preferred means of communication for most rural and urban respondents in both states, but they stressed that the frequency of announcements and jingles targeted at childhood vaccination should be increased. More rural caregivers from Bauchi State, compared to Cross River State, preferred the radio as a means of communicating childhood vaccination messages. The availability of several community radio stations in Bauchi, where the local language (Hausa) was used to deliver information, and poor power supply, made the radio (which could use batteries) more preferred among rural caregivers.

#### Caregivers’ preferred information sources

In both States, but particularly in Bauchi, traditional and religious leadersare highly respected. One of the caregivers from Bauchi explained why traditional institutions were strong and was a preferred source of information:

*“In Bauchi State*, *most of us are Muslims and our traditional leaders are also Muslims so whatever they say stands and no one dare disobeys the traditional leader especially*. *So for us*, *if the Emir is for vaccination and he sends the town announcer to announce it or he tells us himself*, *everyone must obey*. *In other non-Muslim states there may be disagreements between the traditional and religious leaders*.*”*(Caregiver, Bauchi, urban site.)

#### Preferred timing, frequency and content of vaccination information

Caregivers noted that the timing and frequency of vaccination information was also important: Most caregivers wanted more detailed vaccination messages delivered more frequently at the right time and not limited to campaigns.

*“We tend to hear about immunization only when there is a polio or measles campaign*. *After that no one hears anything again till another one is coming up so people may forget or think that is the only vaccination their children should receive*. *I think that they should emphasize that children should continue their immunization at the health facilities after the campaign*. *These campaigns bring a lot of confusion*.*”*(Caregiver, Cross River, urban site)

#### Preferred targets for childhood vaccination information

Many caregivers in Bauchi and a few from Cross River were of the view that targeting men should also be considered when delivering vaccination messages because most women do not take decisions regarding their children without partner consent. Approval from men ensured that necessary arrangements for vaccination, including transportation to the health facility, were made:

*“For us here*, *if our husbands approve immunization then we do not have a choice because he will provide transport and ensure that we take the children for immunization*. *A woman cannot just get up on her own and carry her child for immunization in our community*. *If you are caught you may be sent packing from your marital home and he will take a new wife*. *Even for a widow*, *a man in the family must give approval first*. *This is why I am saying men should be targeted for information at the right time*.*”* (Caregiver, Bauchi, urban site.)

Most rural caregivers suggested that vaccination messages and announcements should be delivered during news broadcasts because men mainly listened to the news, and that this should be done in the local language. Another caregiver stated that vaccination messages delivered in mosques should be given immediately after prayers to capture men’s attention.

### Health workers’ perceptions and experiences of factors affecting childhood vaccination communication

#### a) Attitudes at community level

Health workers in both states identified the attitudes of community members as responsible for vaccination rejection or resistance in certain communities. According to some health workers, certain community members in Bauchi and Cross River prevented health workers from gaining access to their communities, and this prevented them from delivering vaccination messages. In other communities, health workers said that they were sometimes physically attacked, especially when community members felt they had other pressing needs that were not being addressed by the government, such as good road networks, health care, schools for their children and employment.

One health worker noted that two forms of resistance could exist in a community: pocket resistance, where some families within a community refused vaccination; and block resistance, where an entire community refused vaccination. Some reasons cited as contributing to resistance included: misconceptions and rumours linked to religious beliefs, scepticism surrounding the polio vaccine and the perceived marginalization of hard-to-reach communities.

One health worker from Bauchi described how vaccine refusals were more common in the context of polio campaigns and less common in relation to routine immunization in health facilities. This health worker suggested that house visits during campaigns were viewed with more suspicion than vaccines delivered at clinics. This problem was sometimes addressed using community dialogues involving relevant community stakeholders, most often religious or traditional leaders. One religious leader noted that resistance was often linked to religious or cultural beliefs, but that community dialogue usually yielded results.

#### b) Poor interpersonal communication skills among health workers

Various factors were linked to poor communication skill among health workers. In both States, many health workers reported that they received little training in communication. Most health workers in Cross River and a few from Bauchi reported that their only training in communication was received while in college. They pointed out that most workshops and training sessions on the provision of immunization services paid very little attention to the acquisition of communication skills and focused more on technical components of the programmes. Health workers argued that this may have decreased their ability to manage issues of resistance and deliver vaccination messages effectively.

*“In most workshops I attend*, *the emphasis is usually on the vaccines to be administered*, *site of administration*, *storage and maintenance of vaccine potency*, *while communication is usually limited to the types of communication that can be used and the content to mothers bringing their wards for immunization*. *The actual act of communication is not demonstrated*. (Health worker, Cross River, urban site)

One senior healthworker indicated that she had received training on vaccination communication, including the different methods that could be used in various settings, but that this training had taken place four years previously.

#### c) Lack of motivation

Respondents identified a number of factors that undermined their motivation to communicate effectively with parents and communities. During the delivery of communication interventions, some health workers in Bauchi mentioned instances where they were embarrassed by community members who felt that the health worker was shabbily dressed. Consequently, these health workers were not allowed into their homes and were refused access to the children. As mentioned, there were also instances where health workers were attacked by community memberswhen visiting resistant homes or communities.

In Cross River, health workers reported that they had to travel for long distances to remote communities to deliver vaccination services. They also described instances when they were involved in accidents on these trips. They felt that the government rarely paid attention to these challenges, and that they received poor remuneration in relation to the risks involved. They also described how they sometimes had to use personal funds for communication activities but that these funds were rarely reimbursed. These problems discouraged healthworkers and contributed to poor delivery of vaccination information:

*“I trekked a very long distance with vaccines on my head because there were no roads to the community*. *I have even walked through a forest all alone*. *I went through the hills*, *rock and had to cross through this rope bridge*. *I had to cross a deep river once which reached my stomach and it was a fast-flowing river*. *The risks involved can’t be compared to the stipend these [workers] are paid*.*”*(Health worker, Cross River, rural site.)

#### Comparing health workers’ and caregivers’ views on useful channels for vaccination communication

Table 2 compares the preferred sources mentioned by health workers and caregivers. Health workers identified several channels of vaccination information which they felt were useful for caregivers. The most commonly mentioned sources for rural communities were town announcers and traditional and religious leaders whom health workers believed to be useful because they were accessible and acceptable to people. The health workers opined that mothers usually felt important when health workers visited their homes, and that the women usually opened up and asked questions which they did not ordinarily ask in the clinic. These visits also created opportunities for one-to-one interaction and for health workers to provide caregivers with information on both vaccination and other child survival strategies. However, few caregivers mentioned home visits and outreaches conducted by health workers as important and useful. Health workers and caregivers agreed that the media was an important vehicle for delivering communication messages (radio in rural settings and television and radio in urban settings). However, text messages and phone call reminders were less popular among health workers due to their experiences of having to fund these from their personal monies.

**Table 2:** A comparison of health workers’ and caregivers’ views regarding preferred sources of childhood vaccination information.

Preferred source of information	Health workers	Caregivers
Town announcers	++	++
Traditional and religious leaders	++	++
Home visits/ outreach	++	+
Media (radio and television)	++	++
Text message reminders, phone calls	-	++
Health workers	+	++
Community members as mobilizers	-	+

Key

^++:^ Expressed by most respondents

^+:^ Expressed by few respondents

-: Not expressed

## Discussion

Effective communication has been identified as having the potential to strengthen routine immunisation uptake[27].Vaccination has been rightly described by Garcia and colleagues as a shared responsibility between parents and health workers and parents are expected to be active participants in the process [28].A critical factor shaping parental attitudes to vaccination is parents’ interactions with health professionals [29, 30]. This interaction depends on the nature of the communication that takes place between caregivers and the providers of care. This study explored some of these interactions through the perceptions and experiences of caregivers and health workers regarding vaccination communication in two different settings in Nigeria: one setting with relatively high vaccination coverage and one with low vaccination coverage, and within urban and rural locations in both States.

The content and intensity of vaccination messages varied within and across states.This study revealed that, vaccination information was generally sparse in the two settings with urban settings generally having more detailed vaccination related information compared to rural settings. This has serious implications, with majority of the population residing in rural communities, a sector which is usually underserved[31].This study has demonstrated that even basic information provided in immunization clinics in rural areas was sparse. These differences may be attributed to the limited number of personnel in the rural facilities and consequent workload. Many rural health workers are given the task of picking up the vaccines and administering the vaccine on the same day, leaving little or no time to deliver health education.This may be one of the contributing factors to the observed difference in fully immunized children of 15.8% and 42.5% in rural and urban settings respectively in Nigeria[3]. However, compared to Cross River State, Bauchi State had relatively more comprehensive health talks on vaccination. This could be explained by intense efforts by health workers to overcome high vaccine-hesitancy and ultimately improve coverage rates in the State.Effective communication requires targeted messages and planning [19, 32, 33] but this variation suggests that this may be lacking.

Caregivers perceived the clinic environment to influence communication. To help ensure the attention of the caregiver, the health care system needs to make sure the clinic environment is conducive to communication activities. This includes; addressing some basic issues like providing sufficient seats and ensuring good ventilation, and reasonable waiting times. Long waiting times have also been cited by other studies as a common barrier to accessing immunization services, including information[6, 34].When the physical environment of the clinic is ignored it can reduce the attention of the caregiver and act as a barrier to effective communication between the health provider and the caregiver as observed in the present study.

Health workers play an important role in maintaining public trust in vaccination[35]so training of health professionals on how to communicate with others is one of the goals of the ‘Teach skills’ category in the COMMVAC taxonomy[22, 36]. Negative attitudes exhibited by either caregivers or health workers can serve as a deterrent to effective communication and subsequently lead to vaccine hesitancy[19, 37, 38]. In this study, we noted that poor attitudes of health workers towards caregivers as well as poor motivation of health workers could be linked to deficient communication skills among health workers. Gaps in communication interventions directed at health workers were reported in an earlier paper were we explored and mapped the various communication interventions directed at health workers in Nigeria[22].This could possibly explain the deficiencies in communication skills by the health workers. Health workers may benefit from training in ways of being receptive to caregivers and being self-confident in communicating [39, 40] to ensure that they provide relevant and comprehensible information in a respectful and culturally sensitive manner[32]. However, high staff turnover can undermine the value of training. Inadequate staffing can also weaken communication (as was observed in most rural facilities in the two States), while improved staffing can potentially enhance communication. This was also noted in another paper that forms part of the COMMVAC2 project [24].

The most commonly mentioned channels for communication of vaccination information by both health workers and caregivers included town announcers, traditional and religious leaders and the media. These interventions were consistent with what is commonly used in Nigeria, according to our earlier study [22].

Secondly, the above mentioned, communication channels were used more often during campaigns with limited use during routine immunization services. If these communication channels are improved in terms of quality and intensity and included as part of routine immunization programme, they have the potential to improve demand for vaccination services in the community.

Interestingly, in both States, health workers and caregivers regarded traditional and religious leaders as a useful and acceptable channel of communication in both rural and urban settings. Community members may, therefore, be more likely to act on information delivered by these leaders compared to those delivered by health workers. Several studies, suggest that involving traditional and religious leaders as part of the immunization programme could improve vaccination communication delivery, may contribute to improving childhood vaccination uptake [41–45]as well as address vaccine refusals [46]. In order to strengthen the routine immunization programme and consolidate on the gains from the polio programme it might be beneficial that they be actively engaged for routine vaccination services. Several opportunities exist to leverage on the polio footprint to strengthen routine immunization communication. The use of credible communication sources found to be useful and acceptable within the community, such as engagement of traditional and religious leaders in routine immunization programmes, could ensure sustained demand for vaccination services within their communities. Town announcers engaged to provide information to the community during campaigns could be engaged to actively remind community members where and when routine immunisation services are being provided along with simple key messages. These channels would enhance community ownership and be more sustainable in the long run. However, polio campaigns, as seen in most vertical programmes, had little effect on routine programmes, as most resources were used for campaign-related activities. We also suggest that these strategies be implemented in the context of evaluation so that their effects can be better understood.

Health workers reported home visits and outreach as a useful medium for communicating with caregivers, while text messages were seen as useful by caregivers. Similar results were also seen in a study carried out in south western Nigeria, where a majority of caregivers reportedly preferred to receive immunization reminders/recall through mobile phone calls or text messages[47]. Additionally, the use of text messaging has been described as an acceptable and effective channel to improve disease prevention in LMICs[48–50]as well as being effective reminders to improve immunization rates in children[51]. However, our study highlights the need to ensure that health workers do not have to cover the expenses associated with text messaging for it to be useful and sustainable.

It is important that the unique context of people be taken into consideration when planning vaccination communication activities. This study demonstrated that while home visits were acceptable to caregivers in Cross River, caregivers in Bauchi were less likely to assent to these visits. Women in Bauchi explained that the men would usually drop the women at the health facility and pick them up once they had finished at the health facility. These women reported that because they were rarely allowed to go out, they saw the home visits as another opportunity to keep them at home and many of them were therefore not supportive of these visits. Visits to the clinic, on the other hand, afforded them the opportunity to meet with and interact with their fellow women, share experiences and learn from them. These restrictions on the movement of women in Bauchi related to religious beliefs in this State, which were different to those in Cross River.

This could be attributed to the prevailing religions of these States, with Bauchi being predominantly Muslim with more restriction of women and Cross River predominantly Christian with more liberty for the women.

Lastly, the influence of context was also seen in the identified targets for vaccination communication. Men were identified as preferred targets for vaccination communication particularly in rural areas of Bauchi, where women were less likely to take decisions on behalf of the family without the consent of a male family member compared to Cross River. This was also the focus of a recent study from Northern Nigeria where health messages were targeted at respected or influential men in order to enable social approval and encourage the use of service. This strategy was seen to produce positive results and accepted by the men in these settings[45].

### Strengths and limitations of the study

A main strength of the study was our inclusion of two Nigerian states and both urban and rural sites in those states, allowing us to gain a more complete picture of vaccination communication issues. Another strength was the iterative and flexible approach we adopted when conducting the interviews. For example, we went back to some respondents for clarifications of certain issues that we felt were not properly addressed in earlier interviews. Our wide use of direct observation of vaccination communication activities as a data collection method also helped us to validate some of the findings of the interviews. A major limitation was that, for security reasons, we were unable to conduct the study in the northern states with the lowest immunization coverage, where coverage rates may be lower. Also, there is likely a degree of selection bias in the focus groups as caregivers who did not (or could not) attend the vaccination clinic did not have the opportunity to participate in the study. Further studies are therefore needed to elicit the views of caregivers who do not attend vaccination clinics regarding vaccination communication. It is important to know how their views differ from those caregivers who did attend. They may also have differed in terms of education levels or other socioeconomic factors.

Another potential limitation was the fact that the study was conducted during the pre-eradication era of polio in Nigeria, when the attention of governments and international agencies was focused primarily on polio eradication.

## Conclusion

Childhood vaccination coverage is low in Nigeria. Effective communication is required to boost coverage and reduce resistance to the uptake of both existing and new vaccines. Communication about vaccination involves more than the message but is also influenced by the environment and the attitudes of the deliverer and receiver.

Differences were observed based on the setting. We also noted that caregivers and health workers were largely in agreement regarding communication channels, but have identified a number of problems and limitations in the way that these channels are being used.

Vaccination communication should, therefore, be designed to address these issues taking cognizant of the differences in setting. To this end, we suggest that a ‘blanket’ approach to delivering vaccination communication is not what people want or need rather communication channels needs to be tailored by setting and recipient. This will require strategic planning of communication messages, creating a conducive environment for communication, addressing issues to enhance the relationship between caregivers and health workers, and paying attention to preferred channels of communication by caregivers and health workers in local, regional and national settings.

### Appendix 1: Focus group discussion guide (care givers)

Your participation in this interview is totally voluntary. Do you have any questions before we begin?

- **Demographic profile**
  ○ **Child:**
    ■ Age:
    ■ Sex
- **Basic information Mother/ Guardian**:
  ■ Age:
  ■ Highest Educational Attainment:
  ■ Relationship to index child: (where appropriate)
  ■ Number of children:
  ■ Language spoken and whether speaks the language that most materials and information are available in

1. **Immunisation profile**
  ■ Immunisation history of the index child–the most recent (get information from the immunization card)
  ■ Immunization and previous immunizations.
  ■ Immunisation history of other children (where relevant)

1. **Knowledge of /vaccination communication interventions**
  ■ What sources of information and / or support have you received related to immunization? (types of media, friends, family)
  ■ What did you think of the information and / or support you received?
  ■ What kinds of information and / or support would you like to receive?
  ■ In what ways would you like to receive this information or support (how would you like to be communicated with)?
  ■ How often do you receive this information?
2. **What do you think is the purpose of vaccination**?
3. **Details of Knowledge received from Communication interventions**
  ■ What have you learnt from the health talks provided by the health care workers in the clinic that you previously did not know?
  - Did you receive any information about side effects of vaccination?
  - Did you receive any information on when children should not be vaccinated? (contra-indications) note false contraindications
  - Were you told when to come back? Were you given a specific date?
4. **Please could you tell us briefly your experience at the Health facility where you take your wards for immunization**?
  **Probes**:
  - Why did you visit the Clinic?
  - Attitudes and behavior of health care providers
  - Physical environment
  - Access
  - Waiting times
  - Quality of the care received
  - Others specify
5. **Recommendations**
  ■ What do you like about how the clinic is run?
  ■ What do you not like about how the clinic is run?
  ■ Would recommend another mother to bring their wards for the services you have received?

### Appendix 2: Interview guide for vaccinators, lay health workers, traditional and religious leaders

Your participation in this interview is totally voluntary. Do you have any questions before we begin?

**Demographic and other Descriptive information**

- Health careworker cadre -
- Place of work and duration of work in that location:

1 **Background information**
  - How did you start working in vaccination/immunisation?
  - Could you tell me about your work here at the clinic?
  - How long have you been working:
    ○ In vaccination delivery
    ○ At this vaccination/Immunization clinic?

**Please describe your role within the vaccination programme /as a vaccinator**

2 **Vaccination training**
  - Can you tell me about the training you received to work on vaccination?
  - What were the components of the training?
  - Did the training include communication strategies for improving immunisation
  - When was the training received?
  - What sort of materials such as manuals, do you have to support your work?
  - During supervision visits, do you receive any support around communication with caregivers?
3 **Introduction to the vaccination activities**
  - Please describe what you usually do when running a vaccination session/ what happens in the clinic on an average clinic vaccination day
  - How the vaccination is organized
  - How many caregivers are usually seen
4 **Communication interventions**
  - What sorts of information do you / your colleagues / the clinic share with caregivers regarding vaccination? [*Note that we want to find out about the content and format of the different interventions*]

**For each intervention, please describe**:

1. The content of the communication interventions
2. The frequency with which it is delivered and the format/s used
3. Who delivers the intervention
4. Who the communication intervention is targeted to
5. Whether the intervention is used in combination with other interventions
6. What vaccine preventable disease is this intervention targeting?

5 **Communication interventions**
  - For the main vaccination communication interventions that are used in your setting, which has worked well?
  - What challenges / problems do you encounter during this delivery of vaccine information?
  - What discourages you? [*Could include job satisfaction*] (inaccessibility, difficult gate keepers, funding)
  - What other issues may be important in implementing (carrying out) vaccination communication interventions?
  - What resources (material/ non-material)areavailable to support these activities?
6 **Views regarding information and communication**
  - How do you feel about giving information during vaccination?
  - Which information do you think is the most important for parents/care givers to know?
  - What do you think is the easiest source of information for parents?
7 **Relations with community groups**
  - Are there structures / committees (Women leaders, ward development committee, traditional leaders group) in the community around the clinic to which you relate / in which you participate?
  - How do you liaise with important groups in the community?
  - Are there important people/ groups I should speak to in the community regarding vaccination and child health?
  - Are there people / groups within your community or you are aware of that are in favour of or against vaccination?

## Supporting information

S1 File Focus group discussion guidefor caregivers. (DOCX)

S2 File Interview guide for vaccinators, health workers, traditional and religious leaderss. (DOCX)

## Abbreviations

COMMVAC: Communicate to Vaccinate
EPI: Expanded Programme on Immunization
LMICs: Low and middle income countries
WHO: World Health Organisation
VPDs: Vaccine preventable diseases
FGD: Focus Group Discussion
