# Supplementary material for: Perceptions and experiences of childhood vaccination communication strategies among caregivers and health workers in Nigeria: A qualitative study
Source: PLoS One. 2017 Nov 8;12(11):e0186733. doi: 10.1371/journal.pone.0186733 (PMC5678719; doi:10.1371/journal.pone.0186733)
Supplement: S1 File — (DOCX) [file pone.0186733.s001.docx]

# Focus group discussion guide (care givers)

Your participation in this interview is totally voluntary. Do you have any questions before we begin?

- **Demographic profile**
  - **Child:**
    - Age:
    - Sex
- **Basic information Mother/ Guardian** :
  - - Age :
    - Highest Educational Attainment:
    - Relationship to index child: (where appropriate)
    - Number of children:
- Language spoken and whether speaks the language that most materials and information are available in

**1. Immunisation profile**

- - - Immunisation history of the index child – the most recent (get information from the immunization card)
    - Immunization and previous immunizations.
    - Immunisation history of other children (where relevant)

1. **Knowledge of /vaccination communication interventions**
   - What sources of information and / or support have you received related to immunization? (types of media, friends, family)
   - What did you think of the information and / or support you received?
   - What kinds of information and / or support would you like to receive?
   - In what ways would you like to receive this information or support (how would you like to be communicated with)?
   - How often do you receive this information?
2. **What do you think is the purpose of vaccination**?
3. **Details of Knowledge received from Communication interventions**

- What have you learnt from the health talks provided by the health care workers in the clinic that you previously did not know?
  - - - Did you receive any information about side effects of vaccination?
      - Did you receive any information on when children should not be vaccinated? (contra-indications) note false contraindications
      - Were you told when to come back? Were you given a specific date?

1. **Please could you tell us briefly your experience at the Health facility where you take your wards for immunization**?

**Probes:**

- Why did you visit the Clinic?
  - - - Attitudes and behavior of health care providers
      - Physical environment
      - Access
      - Waiting times
      - Quality of the care received
      - Others specify

1. **Recommendations**
   - What do you like about how the clinic is run?
   - What do you not like about how the clinic is run?
   - Would recommend another mother to bring their wards for the services you have received?
