# Supplementary material for: Perceptions and experiences of childhood vaccination communication strategies among caregivers and health workers in Nigeria: A qualitative study
Source: PLoS One. 2017 Nov 8;12(11):e0186733. doi: 10.1371/journal.pone.0186733 (PMC5678719; doi:10.1371/journal.pone.0186733)
Supplement: S2 File — (DOCX) [file pone.0186733.s002.docx]

# Interview guide for Vaccinators, Lay Health Workers, Traditional and Religious Leaders

Your participation in this interview is totally voluntary. Do you have any questions before we begin?

**Demographic and other Descriptive information**

- Health care worker cadre -
- Place of work and duration of work in that location:

1. **Background information**

- How did you start working in vaccination/immunisation?
- Could you tell me about your work here at the clinic?
- How long have you been working:
  - In vaccination delivery
  - At this vaccination/Immunization clinic?

**Please describe your role within the vaccination programme /as a vaccinator**

1. **Vaccination training**

- Can you tell me about the training you received to work on vaccination?
- What were the components of the training?
- Did the training include communication strategies for improving immunisation
- When was the training received?
- What sort of materials such as manuals, do you have to support your work?
- During supervision visits, do you receive any support around communication with caregivers?

1. **Introduction to the vaccination activities**

- Please describe what you usually do when running a vaccination session/ what happens in the clinic on an average clinic vaccination day
- How the vaccination is organized
- How many caregivers are usually seen

1. **Communication interventions**

- What sorts of information do you / your colleagues / the clinic share with caregivers regarding vaccination? [*Note that we want to find out about the content and format of the different interventions*]

**For each intervention, please describe:**

1. The content of the communication interventions
2. The frequency with which it is delivered and the format/s used
3. Who delivers the intervention
4. Who the communication intervention is targeted to
5. Whether the intervention is used in combination with other interventions
6. What vaccine preventable disease is this intervention targeting?
7. **Communication interventions**

- For the main vaccination communication interventions that are used in your setting, which has worked well?
- What challenges / problems do you encounter during this delivery of vaccine information?
- What discourages you? [*Could include job satisfaction*] (inaccessibility, difficult gate keepers, funding)
- What other issues may be important in implementing (carrying out) vaccination communication interventions?
- What resources (material/ non-material) are available to support these activities?

1. **Views regarding information and communication**

- How do you feel about giving information during vaccination?
- Which information do you think is the most important for parents/care givers to know?
- What do you think is the easiest source of information for parents?

1. **Relations with community groups**

- Are there structures / committees (Women leaders, ward development committee, traditional leaders group) in the community around the clinic to which you relate / in which you participate?
- How do you liaise with important groups in the community?
- Are there important people/ groups I should speak to in the community regarding vaccination and child health?
- Are there people / groups within your community or you are aware of that are in favour of or against vaccination?
